# Genetic architecture of endometriosis: risk factors, comorbidities and clinical implications

**DOI:** 10.1093/humupd/dmag023

**Published:** 2026-09-04

**Authors:** Grant W Montgomery, Isabelle M McGrath, Michael Toft Overgaard, Mette Nyegaard, Sally Mortlock

**Affiliations:** Institute for Molecular Bioscience, The University of Queensland, Brisbane, Australia; Department of Health Science and Technology, Aalborg University, Aalborg, Denmark; Institute for Molecular Bioscience, The University of Queensland, Brisbane, Australia; Department of Psychiatry, University of Oxford, Oxford, UK; Department of Chemistry and Bioscience, Aalborg University, Aalborg, Denmark; Department of Health Science and Technology, Aalborg University, Aalborg, Denmark; Department of Congenital Disorders, Statens Serum Institut, Copenhagen, Denmark; Institute for Molecular Bioscience, The University of Queensland, Brisbane, Australia; Australian Women and Girls’ Health Research Centre, School of Public Health, The University of Queensland, Brisbane, Australia

**Keywords:** endometriosis genetics, heritability, genetic risk factors, genetic architecture, endometriosis subtypes, comorbid conditions

## Abstract

**BACKGROUND:**

In 1999, Dr Susan Treloar and colleagues conducted a landmark twin study in Australia and reported their estimate of 51% for the heritability of endometriosis. This important result led several groups to begin mapping genetic factors contributing to increased endometriosis risk. Despite early challenges, advances in genome-wide association studies (GWAS) have identified multiple genetic risk factors and some target genes implicated in follow-up studies on genetic regulation of transcription. Access to large publicly available genetic datasets and analysis with endometriosis GWAS results is also providing new opportunities to answer important questions about comorbid conditions associated with endometriosis and their implications for clinical practice.

**OBJECTIVE AND RATIONALE:**

The objective of the review is to summarize the last 25 years of genetic studies in endometriosis, outline contributions to our understanding of the disease, and suggest future directions to accelerate biological insights from genetic studies to improve clinical outcomes.

**SEARCH METHODS:**

A comprehensive review of scientific literature on the genetics of endometriosis was conducted through searches in PubMed and Google Scholar up to June 2026. Search terms included “endometriosis AND (genetics OR GWAS OR genetic risk factors)”, For studies addressing the functional characterization of genetic risk loci, additional searches employed the terms “endometriosis AND (genotype–phenotype associations OR colocalization OR eQTL OR mQTL OR multi omics methods)”. To identify studies examining shared genetic risk between endometriosis and comorbid conditions, the search strategy included “endometriosis AND (genetic correlation OR colocalization OR Mendelian randomisation)”. Publications reporting discoveries related to genetic risk factors for endometriosis and studies interpreting their biological and clinical significance were critically evaluated, and 144 publications were discussed in the review.

**OUTCOMES:**

Discovery of genetic risk factors started slowly and has accelerated in recent years with developments in technology and international collaborations to combine data and increase statistical power. GWAS have mapped 80 genetic risk factors that implicate gene regulation of hormonal targets, development of the reproductive tract, regulation of cell proliferation, and regulation of epithelial cell differentiation. In common with most other complex diseases, effects of individual common genetic risk factors are small. However, several examples demonstrate that small effect sizes are not a good predictor for the impact of drugs developed against genetically validated targets. Genetic risk factors implicate five genes regulating gonadotrophin release and oestrogen action, the major target pathway of current drugs for treatment of endometriosis demonstrating proof-of-principal for biologically meaningful results. Genetic correlation and Mendelian Randomization studies highlight important causal relationships between endometriosis and comorbid conditions including a possible role for testosterone during development and shared genetic risk factors for gynaecological, gastrointestinal, pain, psychiatric, and inflammatory conditions. Understanding causal relationships between endometriosis and related conditions will aid clinical management and more personalized treatments.

**WIDER IMPLICATIONS:**

Genetic studies provide novel insights into endometriosis pathogenesis and associations with related comorbid conditions. Genetic factors modifying gene regulation and disease risk likely act in specific cell types, and access to datasets from genetically informed cell-based models, single-cell and spatial omics data are needed to accelerate progress. Future studies should address critical questions of heterogeneity and disease subtypes, expand the search for genetic risk factors to non-European populations, evaluate the role of rare and structural variants, and better integrate data from functional, genomics, genetics, and clinical studies to reduce diagnostic delay, develop novel treatment strategies, and translate discoveries into personalized management strategies for affected individuals.

**REGISTRATION NUMBER:**

N/A

## Graphical Abstract

**Graphical Abstract:**
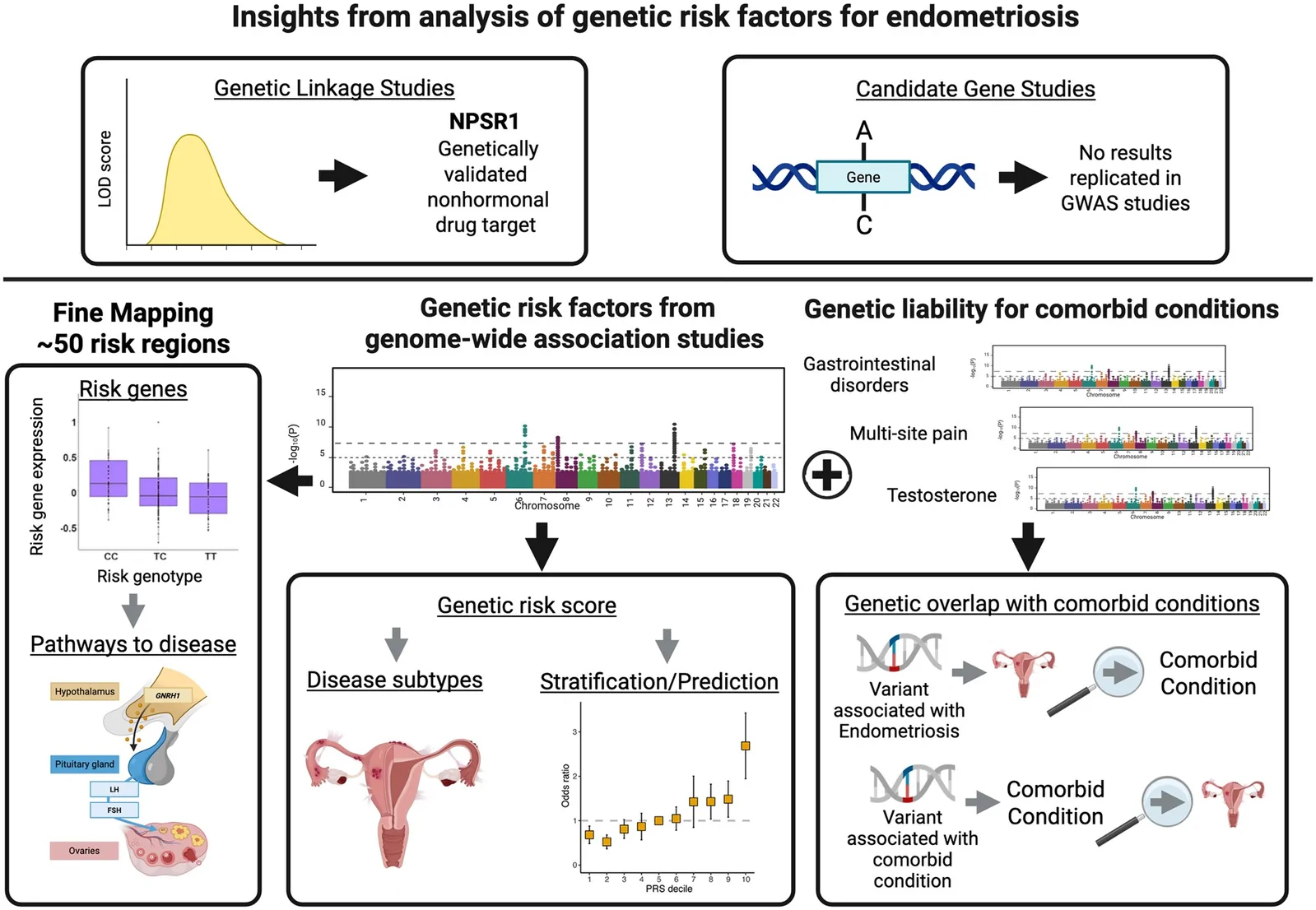
Contributions of genetic studies to understanding risk factors for endometriosis. Created in BioRender. Montgomery, G. (2026) https://BioRender.com/sc7a9p2

## Introduction

In 1999, Dr Susan Treloar, Professor Nick Martin and colleagues published their landmark twin study reporting the first estimate for the heritability of endometriosis (Treloar *et al.*, 1999). Their estimate of 51% was an important result, leading several groups to begin mapping the genetic factors contributing to increased endometriosis risk. Gene mapping efforts gained momentum as methods for low-cost genotyping and gene regulation studies improved with advances in technology and computing power. In 2008, the International Endometriosis Genetics Consortium (IEGC) was formed to combine multiple datasets, increase power for discovery and speed up progress (Painter *et al.*, 2011; Rahmioglu *et al.*, 2015; Montgomery, 2020b). The most recent IEGC study (Rahmioglu *et al.*, 2023) reported 42 genomic regions significantly associated with endometriosis risk.

Success in follow-up and translation of discoveries from the genetic studies has been mixed. The small effect sizes of individual risk loci and difficulty in translating mapped variation in common complex diseases to functional pathways have slowed progress. Although limited in scope, follow-up studies have identified target genes influencing disease biology (Mortlock *et al.*, 2020, 2023; Rahmioglu *et al.*, 2023) and begun to identify novel drug targets (Tapmeier *et al.*, 2021). At the same time, analyzing large publicly available datasets including the UK Biobank, FinnGen, and extensive results from large genetics consortia provides new opportunities to answer important questions about comorbid conditions associated with endometriosis and their implications for clinical practice (McGrath *et al.*, 2023c; Petraglia *et al.*, 2026).

Despite this progress, results from genetic studies are not widely applied in much of the research on disease causation, heterogeneity and clinical trials. Some commentators have questioned the value of genetic studies because known genetic risk factors explain such a small fraction of disease variance (Endometriosis Initiative, 2024) or lack mechanistic links between risk variants and disease pathophysiology (Marquardt *et al.*, 2023). The aims of this review are to summarize the last 25 years of genetic studies in endometriosis and address these important issues, including the proportion of disease variance explained by genetic risk factors, the challenges in establishing direct links between genetic associations and disease pathophysiology, and current understanding of the genetic architecture underlying endometriosis and its common comorbid conditions. In addition, this review seeks to highlight future directions to better integrate genetic studies with cell-based models, functional studies, and clinical research to generate deeper biological insights and improve clinical outcomes.

## Methods

A comprehensive review of the scientific literature on the genetics of endometriosis was undertaken using systematic searches of the MEDLINE (PubMed) database hosted by the National Center for Biotechnology Information and Google Scholar, covering publications available up to June 2026. Peer-reviewed research articles and reviews were identified using the search terms “endometriosis AND (genetics OR GWAS OR genetic risk factors)”. For studies addressing the functional characterization of genetic risk loci, additional searches employed the terms “endometriosis AND (genotype–phenotype associations OR colocalization OR eQTL OR mQTL OR multi omics methods)”. To identify studies examining shared genetic risk between endometriosis and comorbid conditions, the search strategy included “endometriosis AND (genetic correlation OR colocalization OR Mendelian randomisation)”.

Primary search results were supplemented by targeted searches for publications addressing the genetic architecture of complex diseases, as well as manual screening of reference lists from key review articles and original research papers. Inclusion was mainly restricted to primary peer-reviewed research articles published in English. Selected review articles were also included where they provided important conceptual insights or synthesized substantial bodies of earlier work with limited direct relevance to the current discussion, such as early candidate gene studies. Publications reporting discoveries related to genetic risk factors for endometriosis and studies interpreting their biological and clinical significance were critically evaluated and discussed.

## The heritability of endometriosis

Evidence for a genetic influence on endometriosis risk had been accumulating prior to the landmark study by Treloar *et al.* (1999). There is an increased incidence of endometriosis among first-degree relatives of affected individuals in both hospital and population samples, although many of these studies were limited by sample size and potential ascertainment bias (Ranney, 1971; Simpson *et al.*, 1980; Lamb *et al.*, 1986; Coxhead and Thomas, 1993; Moen and Magnus, 1993; Kennedy *et al.*, 1995). To quantify the genetic contribution to endometriosis risk, Treloar and colleagues conducted a twin study comparing disease concordance between monozygotic (MZ) and dizygotic (DZ) twins. Their analysis of Australian twin data revealed a significantly higher concordance rate among MZ twins, with a heritability estimate of 51%. A subsequent study in Sweden yielded a comparable heritability estimate of 47% (Saha *et al.*, 2015). Both estimates suggest that genetic factors account for approximately half of the variation in endometriosis risk. These estimates of heritability are similar to those reported for other reproductive traits, including age at menarche, age at menopause, and uterine fibroids (McGrath *et al.*, 2021), supporting a substantial genetic component to reproductive health.

## Genetic and genomic contributions to endometriosis risk

### Mapping genetic risk factors

Early mapping studies employed two main strategies: linkage analysis, which had proven effective in identifying mutations for rare diseases (Treloar *et al.*, 2005), and candidate gene studies (Montgomery *et al.*, 2008; Rahmioglu *et al.*, 2015). Both approaches were feasible given the genetic marker technologies available at the time but ultimately had limited success. While linkage studies successfully mapped loci underlying monogenic disorders, they mostly failed when applied to common diseases because the individual causal variants typically exerted small effects, making them difficult to detect through co-segregation in pedigrees (Sidorenko *et al.*, 2024). Candidate gene studies also failed in part due to the small effect sizes of individual variants. Few reported candidate gene associations with endometriosis or any complex diseases replicated in larger, more robust genome-wide association studies (GWAS) (Duncan *et al.*, 2019; Montgomery, 2020a). Most studies relied on small sample sizes and failed to adequately correct for multiple testing, which undermined the reliability of their findings. Applying more stringent multiple testing corrections, equivalent to those used in GWAS, would have reduced false positive results (Montgomery, 2020a).

Linkage studies in endometriosis identified two genomic regions possibly associated with endometriosis. A region on chromosome 10q26 showed evidence of linkage (Treloar *et al.*, 2005) but subsequent extensive fine mapping across this region did not support the association (Painter *et al.*, 2011). Linkage analyses conducted in families with three or more endometriosis cases identified a high-penetrance susceptibility locus for endometriosis on chromosome 7p (Zondervan *et al.*, 2007).

Genetic and functional follow-up identified the neuropeptide S receptor 1 (*NPSR1*) gene and the encoded protein Neuropeptide S (NPS) as a potential nonhormonal target for the treatment of endometriosis (Tapmeier *et al.*, 2021). Follow-up studies suggest NPS may induce cell invasion and actin cytoskeleton remodelling (Prisinzano *et al.*, 2024) and support a role for *NPSR1* antagonists in blocking peripheral inflammation (Berger *et al.*, 2025).

### Genome-wide association studies

The first strong evidence for genetic risk factors associated with endometriosis was reported in a Japanese study (Uno *et al.*, 2010). This was followed by a series of GWAS conducted in different populations, including European (Painter *et al.*, 2011), USA (Albertsen *et al.*, 2013), Icelandic (Steinthorsdottir *et al.*, 2016), Polish (Sobalska-Kwapis *et al.*, 2017), and Taiwanese (Sheu *et al.*, 2024) cohorts. To enhance statistical power and identify robust associations, meta-analyses combining data from multiple populations have been conducted (Nyholt *et al.*, 2012; Rahmioglu *et al.*, 2012, 2023; Sapkota *et al.*, 2017; Koller *et al.*, 2026).

A meta-analysis of data from 60 674 cases and 701 926 controls of European and East Asian descent identified genome-wide significant association with 42 genomic regions (Rahmioglu *et al.*, 2023), of which 31 regions were novel. The study replicated 11 of 15 regions previously associated with endometriosis (Uno *et al.*, 2010; Painter *et al.*, 2011; Nyholt *et al.*, 2012; Albertsen *et al.*, 2013; Sapkota *et al.*, 2015a, b, 2017; Steinthorsdottir *et al.*, 2016). Additional genetic risk factors continue to be identified with increasing sample size. The most recent meta-analysis analyzed data from ∼1.4 million women, including 105 869 cases from diverse ancestries (Koller *et al.*, 2026). This cross-ancestry analysis identified genome-wide significant association for 80 independent variants, including 37 novel results (Koller *et al.*, 2026). Some differences in significant results between published studies are likely due to case ascertainment. Genetic association with *IL1A* (Chr 2p13) and *FN1* (Chr 2q35) regions was not replicated in the recent International Endometriosis Genetics Consortium (IEGC) study (Rahmioglu *et al.*, 2023). This was attributed to heterogeneity in case ascertainment across studies with a higher proportion of less severe cases in the IEGC study (Rahmioglu *et al.*, 2023). Effect sizes for the risk variants in the *IL1A* and *FN1* regions are higher for severe disease, consistent with previous results (Sapkota *et al.*, 2017). Both these regions again showed genome-wide significant association in the most recent meta-analysis (Koller *et al.*, 2026).

The association of rs10965235 on chromosome 9p21, initially reported in the Japanese population (Uno *et al.*, 2010), appears to be population specific since rs10965235 is not polymorphic in European populations (Painter *et al.*, 2011). Associations with the *RND3-RBM43* region at chromosome 2q23.3 (Albertsen *et al.*, 2013) and *TTC39B* at chromosome 9p22 (Steinthorsdottir *et al.*, 2016) reported in earlier studies were not replicated in subsequent meta-analyses (Sapkota *et al.*, 2017; Rahmioglu *et al.*, 2023) also suggesting potential population differences, although association in the *TTC39B* region identified in the Icelandic population did replicate in samples from the Danish National Birth Cohort (Steinthorsdottir *et al.*, 2016). A recent GWAS in the Taiwanese population identified two novel loci, C5orf66/C5orf66-AS2 (5q31.1) and the *STN1* (STN1 Subunit of CST Complex, 10q24.33) not previously reported in European studies (Rahmioglu *et al.*, 2023). Although cross-ancestry analysis increased the discovery of genome-wide associations in the most recent analysis, no ancestry specific variants were identified across population groups (Koller *et al.*, 2026). However, heterogenous effects across populations were reported for six regions (*WNT4*, *GREB1*, *KDR*, *SYNE1*, *FSHB*, and *VEZT*) (Koller *et al.*, 2026).

### The genetic architecture of endometriosis

The genetic architecture for a disease or trait depends on how many genetic variants are involved, how common they are, and how much they affect the trait. Studies using GWAS have shown that genetic risk for most common diseases, including endometriosis, results from the additive effects of many variants each with small effects (Visscher *et al.*, 2021; Rahmioglu *et al.*, 2023). There is little evidence yet that single gene mutations with large effects play a role in endometriosis (Sapkota *et al.*, 2017). As seen for other diseases (Agrawal *et al.*, 2025), many additional common risk variants will be discovered in future studies with larger samples.

Genetic studies remain heavily biased towards European populations owing to historical data availability, and findings do not always generalize well to other ancestries. The recent cross-ancestry analyses demonstrates heterogeneity in effect sizes, highlighting both shared biology and potential ancestry-specific genetic effects (Koller *et al.*, 2026). In addition, extending studies into non-European ancestries with detailed genetic and phenotypic data and in diverse countries with different healthcare systems may provide unique information about the various environmental factors that affect disease risk and their relation to genetic risk factors (Peterson *et al.*, 2019). It is expected this will be an active area of research for endometriosis in the future (Gajbhiye *et al.*, 2021; Bhurke *et al.*, 2022).

### Variance explained

The IEGC meta-analysis found 42 regions in the genome linked to endometriosis (Rahmioglu *et al.*, 2023). The strongest genetic variants or lead single nucleotide polymorphisms (SNPs) from each region explained only 1.62% of the overall variation in symptoms, or about 3% of the genetic risk, assuming the heritability is 50% (Rahmioglu *et al.*, 2023). There are secondary independent signals in several regions, and when all 49 independent lead SNPs are included, the variance explained increased slightly to 1.98% of phenotypic variance and ∼4% of genetic risk (Rahmioglu *et al.*, 2023).

As noted by others (Marquardt *et al.*, 2023; Endometriosis Initiative, 2024), the genome-wide significant lead SNPs explain only a small proportion of the variance in endometriosis risk. However, when looking at all common SNPs across the genome, we get a different picture. This broader estimate, called SNP heritability, includes the small contributions to endometriosis risk from many SNPs across the genome, not just the small number of genome-wide significant SNPs from our current GWAS of modest size. The SNP heritability for endometriosis explained 27% of disease risk in the first European GWAS (Painter *et al.*, 2011) and has stayed fairly consistent in later studies (Lee *et al.*, 2013, 2017). Common SNPs together explain about 53% of the genetic risk estimated from twin studies, and 26% of the overall variation in endometriosis.

### Genetic profiles and disease subtypes

These genetic estimates are based on treating all endometriosis cases as one group. However, endometriosis is a heterogeneous disease with highly variable clinical presentation. A key question is whether it is a single disease with variable symptoms or has distinct subtypes. The disease has many observable heterogenous features and patients experience variable symptoms; severe menstrual pain, subfertility or both, and a range of co-occurring conditions that significantly impact quality of life (Horne and Missmer, 2022; As-Sanie *et al.*, 2025). Endometriosis in the pelvis can be classified as ‘superficial’ (<5 mm penetration), ‘deep’ (>5 mm penetration), or ovarian (cysts on the ovaries), may be associated with pelvic adhesions, and extra-pelvic endometriosis has been reported in some cases (Giudice, 2010; Horne and Missmer, 2022). Surgical excision/ablation of the lesions is sufficient to relieve symptoms in some, but not others.

Disease subtypes could relate to the physiological, symptomatic, or molecular features of the endometriosis lesions. Important evidence from genetic studies supporting different disease subtypes is that genetic risk is higher in more severe cases. For example, in cases classified as Stage III/IV by the American Fertility Society (AFS), the SNP heritability was 0.34, twice the estimate of 0.15 seen in milder Stage I/II cases (Painter *et al.*, 2011). This increased genetic risk associated with more severe disease is not uniform. Effect size estimates for some lead SNPs show much greater effects in severe (AFS Stage III/IV) cases compared with all cases, with a trend for SNPs with larger effect sizes to show the biggest differences.

Genetic differences in disease severity, as defined by AFS scores, have been investigated using available datasets that integrate both genotype data and AFS classifications. However, the AFS classification has limitations, and there is a growing shift towards alternative frameworks that better capture the heterogeneity of endometriosis phenotypes. For example, the most recent GWAS meta-analysis identified five regions that were genome-wide significant only in clinically defined endometriosis cases (*GRB14* on chr2, *CABS1* on chr 4, *RNA5SP375* on chr 12, *RP11-467I17.1* on chr 16, and *ZHX3* on chr 16) and one region (*LINC00485* on chr 12) only significant in self-reported cases suggesting phenotypic definitions of endometriosis influence the specificity of gene discovery (Koller *et al.*, 2026). Increasing the availability of datasets with detailed phenotype and genetic data will facilitate future studies to understand the genetic architecture of specific disease subtypes and the heterogeneity of endometriosis.

### Genetic risk prediction

There is increasing interest in using genetic variants associated with a condition for patient stratification or risk prediction in clinical settings. This risk can be represented as a score, termed a genetic risk score (GRS) or polygenic risk score (PRS). In its simplest form, a GRS is calculated as the sum of the effects for the risk-increasing alleles for an individual, in some cases weighted by the effect of each allele estimated in GWAS. Improving the clinical risk assessment of endometriosis is important as the symptoms of endometriosis can be non-specific, there is a long diagnostic delay, and the definitive diagnosis of laparoscopic surgery is invasive.

Kloeve-Mogensen *et al.* (2021) evaluated the utility of a 14-SNP genetic risk score (GRS) for endometriosis in a combined Danish cohort (389 cases, 664 controls) and the UK Biobank (2967 cases, 256 222 controls). The GRS was significantly associated with endometriosis diagnosis, yielding an AUC of 0.64 in the Danish cohort. Individuals in the top 10% of the GRS distribution had a threefold increased risk compared to those with average scores. Notably, ovarian endometriosis exhibited the highest AUC in both cohorts, suggesting either a greater genetic burden or better predictive performance of the GRS for this subtype. Conversely, the GRS showed poor predictive value for adenomyosis, supporting the notion that these are genetically distinct conditions.

In a more recent analysis, McGrath *et al.* (2025) derived a GRS from summary statistics of 28 382 independent endometriosis cases. Applying this in the UK Biobank, the mean GRS was higher in cases compared to controls, with an AUC of 0.61. Compared to individuals at the 50th percentile, those in the 1st centile had an odds ratio (OR) of 0.38, while those in the 100th centile had an OR of 3.67. In the recent cross-ancestry study (Koller *et al.*, 2026), individuals of European ancestry in the highest GRS quartile had an increased odds of endometriosis (OR = 2.19, 95% CI = 2.07–2.32) compared with individuals in the lowest quartile. GRS demonstrated cross-ancestry transferability but with reduced predictive performance across populations reflecting the genetic distance between European and the other population groups (Koller *et al.*, 2026), emphasizing the need for more diverse datasets.

As noted earlier, evidence supports variation in genetic architecture by disease stage with a higher proportion of variance explained by the GRS for severe disease (Painter *et al.*, 2011; Rahmioglu *et al.*, 2023). The ability to predict disease using genetic risk factors is strongest for severe (Stage IV) cases, but the GRS for mild cases does provide some prediction across samples (Sapkota *et al.*, 2015a). GRS estimates show high technical reproducibility using established genotyping platforms and quality control pipelines (Lin *et al.*, 2026) but prediction accuracy is limited for most common diseases (Zeng and Visscher, 2025). For endometriosis, the current AUC of 0.61 limits clinical utility (McGrath *et al.*, 2025; Koller *et al.*, 2026). It may be useful for risk stratification when combined with clinical features or other risk factors. In future, predictive power may also increase from larger GWAS datasets, stratification by subtypes, inclusion of rare variants, and enhanced statistical methodologies incorporating functional evidence into the risk estimates (Zeng and Visscher, 2025; Wainschtein *et al.*, 2026).

### Other sources of DNA variation

Identifying coding variants that increase risk of endometriosis and modify protein function through amino acid substitutions, altered stop signals, or variable splicing is a direct approach to discover functionally significant risk genes. While imputation methods have improved genome coverage for common variants in coding regions, they have limitations in detecting rare variants. This is because the accuracy of single-variant tagging declines markedly for variants with minor allele frequencies (MAFs) less than 5%.

In an early study, coding variants from an Illumina exome-array were genotyped directly in 7164 cases and 21 005 controls of European ancestry to evaluate the role of protein-modifying variants (Sapkota *et al.*, 2017). After quality control, single-variant analysis included 23 755 rare variants (MAF < 1%) and 8858 low-frequency variants (1%<MAF < 5%). This approach was not fruitful: one low-frequency variant (rs1801232) reached statistical significance in the discovery set following correction for multiple testing but failed replication in an independent sample, indicating much larger cohorts and improved rare variant typing will be necessary.

Potential high-risk variants have been reported from exome sequencing in two families each with four affected individuals, and in an endometriosis case study (Peng *et al.*, 2021; Nousiainen *et al.*, 2023; Santin *et al.*, 2023; Kina *et al.*, 2024). In one family from Finland (Nousiainen *et al.*, 2023), rare segregating variants that might predispose to endometriosis were reported in *FGFR4* (Fibroblast Growth Factor Receptor 4), *NALCN* (Sodium Leak Channel, Non-Selective), and *NAV2* (Neuron Navigator 2). Rare variants in two different genes, *TNFRSF1B* (TNF Receptor Superfamily Member 1B) and *GEN1* (GEN1 Holliday Junction 5' Flap Endonuclease) were reported in a second family from Türkiye (Kina *et al.*, 2024). Sequencing studies in two small cohorts identified five rare protein-coding variants and two novel mutations in *DYSF* (Dysferlin) in a Chinese population (Peng *et al.*, 2021) and several rare variants or private mutations in endometriosis cases from Italy (Santin *et al.*, 2023). Validation of a role for any of these variants in endometriosis will require functional studies or possible replication in independent samples.

While whole-genome sequencing (WGS) remains expensive for the large-scale studies required to reliably identify rare variants associated with disease, data for endometriosis cases are becoming available through resources such as the UK Biobank, OurFutureHealth (Cook *et al.*, 2025) and AllofUS (Kumar *et al.*, 2025). This will enable a more comprehensive evaluation of rare variants for endometriosis. Recent analysis of WGS data in the UK Biobank helped to quantify the contributions of both rare and common variants for 34 complex traits (not including endometriosis) (Wainschtein *et al.*, 2026). WGS captured approximately 88% of the estimated heritability for these traits, with 20% attributed to rare variants and 68% to common variants. The study provides a good estimate of the contribution of rare variants to genetic variation and demonstrates that the heritability for some traits can be fully accounted for by the observed variants from WGS (Wainschtein *et al.*, 2026).

Another relevant source of DNA variation is somatic mutations. They arise in individual cells from errors in DNA replication or exposure to exogenous mutagens (Forsberg *et al.*, 2017). Somatic mutations are not inherited but represent important genomic alterations that may interact with germline variation to influence gene regulation and cellular fate. Their frequency is a function of the number of cell divisions since conception and the mutation rate per division (Forsberg *et al.*, 2017). High rates of proliferation occur in the endometrium, a key disease-relevant tissue in endometriosis. In this tissue, the functionalis layer undergoes cyclical shedding and regeneration during each menstrual cycle and may be one source of cells that initiate lesions (Ochoa Bernal and Fazleabas, 2024). The high rates of cellular turnover increase the likelihood of mutations occurring, and somatic mutations have been observed in both eutopic endometrium and in endometriotic lesions (Anglesio *et al.*, 2017; Suda *et al.*, 2018; Lac *et al.*, 2019; Koppolu *et al.*, 2021), with a predominance in epithelial cell populations.

Somatic mutations are likely to be functionally relevant to disease pathogenesis. Mutations in known cancer driver genes occur more frequently than mutations in non-driver genes (Anglesio *et al.*, 2017; Koppolu *et al.*, 2021), and alterations in cancer driver genes may provide a selective advantage to cells with enhanced ability to survive, implant and proliferate. Accumulating evidence supporting their functional significance underscores the need to investigate the relationship between common germline variants and somatic mutational processes in endometriosis.

## Identifying risk genes and pathways through functional studies

A major challenge of genetic mapping in complex diseases lies in the fact that most lead SNPs identified by GWAS are located in non-coding regions of the genome. Most non-coding disease risk variants likely modify local regulation of a critical gene or genes (defined as risk genes for this review) to modify disease risk (Mostafavi *et al.*, 2023). There are usually multiple genes in the vicinity of each lead SNP and no easy way to identify the critical risk gene or genes. Multiple methods must be applied combining genetic and functional studies (Qi *et al.*, 2024). For a limited number of traits, a high proportion of GWAS ‘hits’ are situated near well-recognized candidate genes. Examples include age at menopause and dizygotic twinning (Kentistou *et al.*, 2024; Montgomery, 2024), where regulatory pathways are well studied and critical genes have been clearly identified. Despite the numerous genetic markers associated with human traits and diseases, slow progress in defining causal genes and pathways continues to limit translation of GWAS discoveries. Endometriosis is no exception, but some insights are beginning to emerge.

### Candidate genes

Some GWAS signals for endometriosis map close to strong candidate genes (Fung *et al.*, 2018; GTEx Consortium, 2020; Mortlock *et al.*, 2020, 2023). Endometriosis is an oestrogen dependent disease and five independent signals for endometriosis are located in or near *ESR1* (Estrogen Receptor 1 gene) on chromosome 6 (Rafnar *et al.*, 2018; Marla *et al.*, 2021; Rahmioglu *et al.*, 2023; Koller *et al.*, 2026). These span the region from *CCDC170* (Coiled-Coil Domain Containing 170) immediately upstream of *ESR1* to *SYNE1* (Spectrin Repeat Containing Nuclear Envelope Protein 1) (Sapkota *et al.*, 2017; Marla *et al.*, 2021). Two high confidence variants within the introns of *SYNE1* and *ESR1* are strongly correlated with variants driving expression of *ESR1* and DNA methylation near *ESR1* and *SYNE1* (Rahmioglu *et al.*, 2023). Risk factors for breast cancer, age of menarche, age at first birth, and uterine fibroids also map to this region but the risk SNPs for endometriosis and other reproductive traits are largely distinct (Rafnar *et al.*, 2018; Marla *et al.*, 2021) suggesting complex regulation of these genes across tissues and cell types.

Another key candidate is *FSHB*, which encodes the β subunit of follicle-stimulating hormone (Sapkota *et al.*, 2017; Rahmioglu *et al.*, 2023). The lead SNP (rs3858429) (Rahmioglu *et al.*, 2023) is highly correlated to variants upstream of *FSHB*. The association signal in this region upstream of the *FSHB* promoter includes four highly correlated SNPs and the region exhibits open chromatin in gonadotrophs in the pituitary gland (McGrath *et al.*, 2021). These SNPs are associated with *in vivo* concentrations of FSH, luteinizing hormone (LH), steroid hormone binding globulin (SHBG), and testosterone (T) (Mbarek *et al.*, 2024) and likely act through effects on hormonal regulation. The common allele (frequency = 0.84) associated with increased FSH concentrations is also associated with multiple reproductive traits and diseases including increased endometriosis risk, shorter menstrual cycles, and increased dizygotic twinning frequency (McGrath *et al.*, 2021).

Interestingly, *ARL14EP* (ADP Ribosylation Factor Like GTPase 14 Effector Protein), a gene that lies immediately downstream of *FSHB*, is also implicated. It is expressed in many tissues including strong expression in immune calls, ovaries, testis, and uterus (McGrath *et al.*, 2021; Rahmioglu *et al.*, 2023; Mbarek *et al.*, 2024). The lead SNP for endometriosis (rs3858429) (Rahmioglu *et al.*, 2023) is highly correlated with a SNP in the 5′ UTR region regulating expression of *ARL14EP* where the common C allele is associated with lower *ARL14EP* expression in testis (GTEx Consortium, 2020). The effector protein encoded by *ARL14EP* regulates immune responses via MHC class II export (Paul *et al.*, 2011) suggesting a possible link between hormone and immune regulation at this locus (Rahmioglu *et al.*, 2023).

### Risk genes supported by functional evidence

A common method of annotating GWAS risk variants is by integration with multi-omics datasets, which provide maps of the genetic regulation of gene expression (eQTLs), methylation (mQTLs), and more recently protein expression (pQTLs). Genetic variation has been shown to influence DNA methylation signals (Min *et al.*, 2021) and contribute to expression differences in a substantial proportion of genes (GTEx Consortium, 2020; Vosa *et al.*, 2021). SNPs associated with variation in methylation or gene expression offer insights into potential risk genes, especially when observed in disease-relevant tissues. However, regulatory variants are widespread in the genome, so overlap in disease-associated SNPs and regulatory signals may be driven by linkage disequilibrium or chance (Liu *et al.*, 2019). Statistical methods can test the alternative models to assess the likelihood of shared causal variants (Wallace *et al.*, 2012; Zhu *et al.*, 2016).

Analysis of genetic effects on gene expression for risk regions in the IEGC meta-analysis was conducted in three datasets for endometrium and uterus, and one large dataset for blood (Rahmioglu *et al.*, 2023). Overlap with eQTL and mQTL datasets (GTEx Consortium, 2020; Mortlock *et al.* 2020, 2023; Min *et al.*, 2021; Rahmioglu *et al.*, 2023) provides increasing evidence for GWAS risk variants regulating expression of risk genes and methylation signals (Table 1). Risk regions often harbor multiple variants, and a single causal SNP can influence the expression of more than one gene, further complicating the identification of causal variants and their target genes. Four regions in the IEGC analysis (Rahmioglu *et al.*, 2023) exhibited multiple independent signals: three regions (*GREB1*/2p25.1, *CDKN2-BAS1*/9p21.3, and IGF1/12q23.2) had two signals each and *SYNE1*/6q25.1 had five independent signals (Rahmioglu *et al.*, 2023). Examples of genetic regulation of risk genes include variants in enhancers or promoters that alter gene expression of one or more candidates, variants modifying hormonal concentrations, or effects on transcript splicing (Fig. 1).

**Figure 1.**
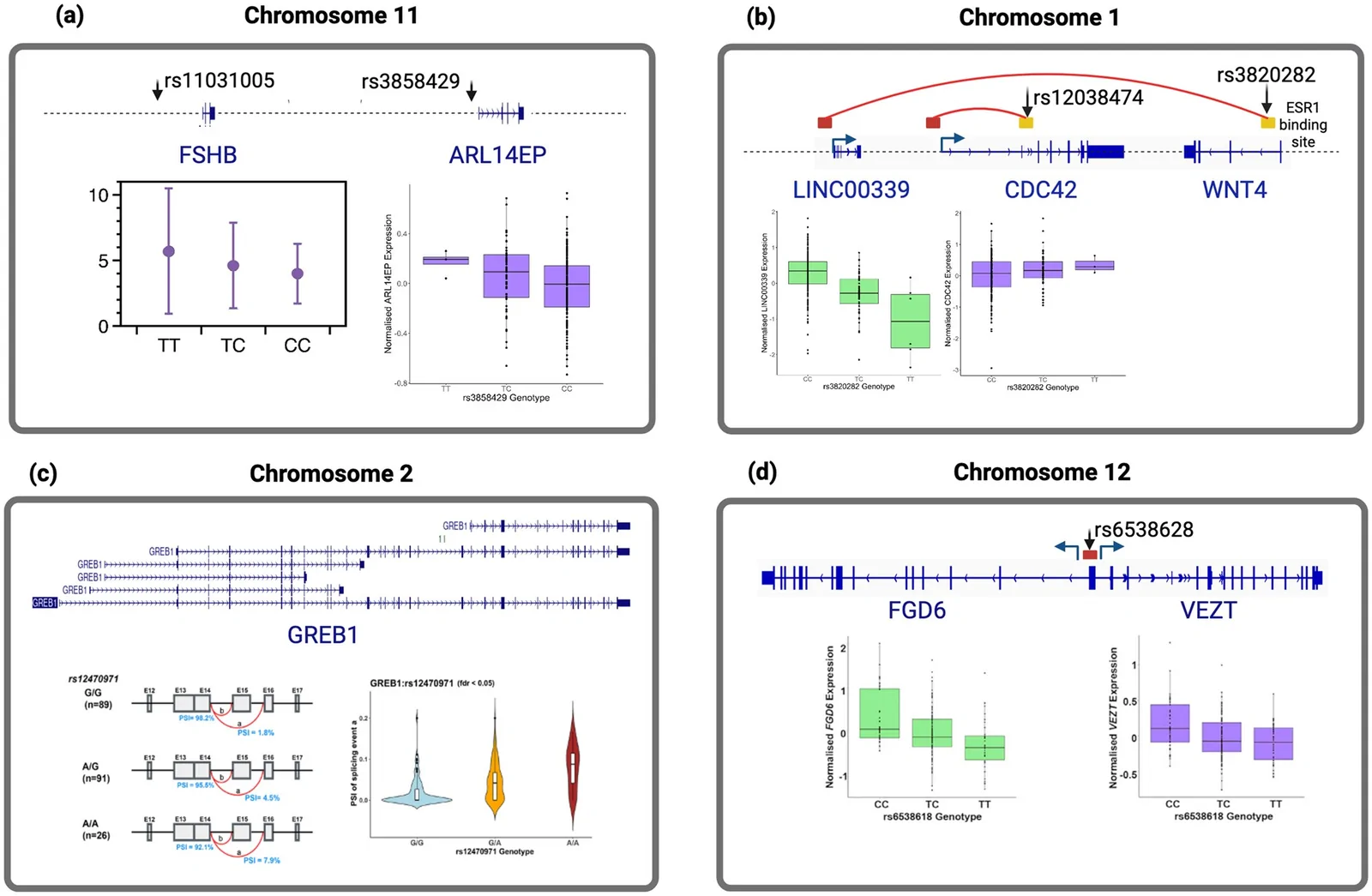
**Genetic risk factors influence functional pathways through different mechanisms**. Examples of different mechanisms for how lead single-nucleotide polymorphisms (SNPs) influence regulation of genes and hormonal concentrations: (**a**) the common alleles of correlated risk SNPs on chromosome 11 associated with increased risk of endometriosis are also associated with an increase in follicle-stimulating hormone (FSH) concentrations and decreased expression of ARF Like GTPase 14 Effector Protein (*ARL14EP)*; (**b**) on chromosome 1, risk SNPs show independent signals increase expression of the long non-coding RNA *LINC00339* and decrease expression of Cell Division Cycle 42 (*CDC42)*; (**c**) on chromosome 2, the risk SNP is associated with alternative splicing of transcripts for Growth Regulating Estrogen Receptor Binding 1 (*GREB1*); and (**d**) the risk SNP on chromosome 12 lies in a bidirectional promoter decreasing expression of both FYVE, RhoGEF and PH Domain Containing 6 (*FGD6)* and Vezatin, Adherens Junctions Transmembrane Protein (*VEZT)*. Created in BioRender. Montgomery, G. (2026). https://BioRender.com/3m1rpt7.

**Table 1.** Endometriosis risk regions from Rahmioglu *et al.* (2023) with evidence^1^ that risk SNPs regulate expression of local genes through expression quantitative trait loci (eQTLs) or DNA methylation quantitative trait loci signals (mQTLs).

Locus	Chr	Lead SNP	RA	Target	Tissue^2^	Gene^3^	SNP	mQTL probe	LD(r2)^4^	LD(D')^4^
*WNT4/1p36.12*	1	rs10917151	A	mQTL	Blood	*WNT4*	rs55938609	cg15582954	0.75	0.96
*GREB1/2p25.1*	2	rs11674184	T	mQTL	EM	*GREB1*	rs28689909^	cg07314298	0.72	0.95
*GREB1/2p25.1*	2	rs11674184	T	mQTL	EM	*GREB1*	rs59129126^	cg16908938	0.75	0.96
*ID4/6p22.3*	6	rs6456259	G	eQTL	EM	*LNC-LBCS*	rs17483277	NA	0.15	0.59
*CD109/6q13*	6	rs4540228	C	mQTL	Blood	CD109	rs2917883^	cg16124188	0.29	1
*HEY2/6q22.31*	6	rs2226158	G	mQTL	Blood	*ESR1*	rs851996	cg18745416	0.41	0.99
*SYNE1/6q25.1*	6	rs71575922	G	mQTL	Blood	*ESR1*	rs3020333	cg22157087	0.33	0.94
*SYNE1/6q25.1*	6	rs71575922	G	mQTL	Blood	*ESR1*	rs862346	cg20893956	0.41	1
*SYNE1/6q25.1*	6	rs71575922	G	mQTL	Blood	*ESR1*	rs1101080	cg04063345	0.83	0.96
*SYNE1/6q25.1*	6	rs71575922	G	mQTL	Blood	*ESR1*	rs1101080	cg15626350	0.83	0.96
*SYNE1/6q25.1*	6	rs71575922	G	mQTL	Blood	*SYNE1*	rs2881823	cg01066157	0.89	0.96
*FAM120B/6q27*	6	rs11756073	A	mQTL	Blood	*LINC00574*	rs1532389^	cg05081416	0.99	1
*7p15.2/7p15.2*	7	rs1451383	G	eQTL	EM	*TRA2*	rs10238290	NA	0.00	0.09
*GDAP1/8q21.11*	8	rs10090060	A	eQTL	Blood	*GDAP1*	rs10283076	NA	0.77	0.97
*GDAP1/8q21.11*	8	rs10090060	A	mQTL	Blood	*GDAP1*	rs4567029	cg23779890	0.77	0.97
*MLLT10/10p12.31*	10	rs10828249	A	mQTL	Blood	*MLLT10*	rs12251016^	cg04231319	0.94	0.98
*FSHB/11p14.1*	11	rs3858429	C	mQTL	Blood	*FSHB*	rs11031040^	cg13280803	1	1
*FSHB/11p14.1*	11	rs3858429	C	mQTL	Blood	*ARL14EP*	rs12271187^	cg26197155	1	1
*HOXC10/12p13.13*	12	rs3803042	A	mQTL	Blood	*HOXC-AS2*	rs3803042^	cg16264616	1	1
*VEZT/12q22*	12	rs12320196	G	eQTL	EM	*VEZT*	rs7296348	NA	0.39	0.99
*SRP14-AS1/15q15.1*	15	rs12441483	C	eQTL	EM	*SRP14*	rs4770	NA	0.37	0.93
*SRP14-AS1/15q15.1*	15	rs12441483	C	mQTL	Blood	*SRP14-AS1*	rs12441483^	cg27155939	1	1
*SRP14-AS1/15q15.1*	15	rs12441483	C	mQTL	Blood	*BMF*	rs7162269^	cg00868766	0.89	0.99
*SRP14-AS1/15q15.1*	15	rs12441483	C	mQTL	Blood	*BMF*	rs7162269^	cg16650630	0.89	0.99
*SKAP1/17q21.32*	17	rs66683298	C	eQTL	EM	*HOXB9*	rs2938475^	NA	0.66	0.92

1 eQTLs and mQTLs with statistical support that the same genetic variant affects endometriosis risk and gene expression or methylation signals.

2 Data sets for gene expression or methylation in endometrium (EM) or blood.

3 Gene regulated by the functional single nucleotide polymorphism (SNP) (eQTLs) or nearest gene to modified methylation signals.

4 Linkage disequilibrium (LD) between the lead and functional SNPs estimated as *r*^2^ or *D*′.

One example of complex effects is the lead SNP rs10917151 on chromosome 1p36 (Rahmioglu *et al.*, 2023) which is highly correlated with rs3820282 in the first intron of *WNT4* (Wnt Family Member 4). This region shows chromatin looping to the promoter of *LINC00339* (Fig. 1) increasing the expression of this long non-coding RNA (Powell *et al.*, 2016). The risk allele (T) for rs3820282 generates a novel enhancer with strong binding for *ESR1* (Zhang *et al.*, 2017). *LINC00339* is now known to be transcribed into a protein, expressed in endometriosis lesions, and has effects on the expression of genes involved in immune defence pathways (Holdsworth-Carson *et al.*, 2021). Direct effects of rs3820282 on *WNT4* expression in human endometrium were not observed (Powell *et al.*, 2016; Mortlock *et al.*, 2020), yet evidence in mice suggests *WNT4* may be regulated by rs3820282: The DNA sequence around rs3820282 is 98% conserved in mice and gene editing to introduce the T risk allele at this site in mice resulted in upregulation of Wnt4 in endometrial stromal cells (Pavlicev *et al.*, 2024). The risk variant suppresses proliferation in the epithelium and enhances pro-implantation/invasion processes in the stroma. A second variant associated with endometriosis in this region lies in an intron of *CDC42*, interacts with the *CDC42* promoter and reduces expression of this gene (Fig. 1) (Powell *et al.*, 2016). Knockdown experiments also suggest the *LINC00339* locus may regulate expression of *CDC42* (Chen *et al.*, 2018; Pavlicev *et al.*, 2024).

The likely causal gene at chromosome 2p25 is *GREB1*, an oestrogen-responsive gene involved in oestrogen receptor transcription, and cell proliferation in breast, prostate, and ovarian cancers (Mortlock *et al.*, 2019; Rahmioglu *et al.*, 2023). Analysis of RNA splicing shows a risk SNP strongly correlated with the lead GWAS SNP alters RNA splicing of *GREB1* in the ovary and endometrium (Fig. 1) (GTEx Consortium, 2020; Yang *et al.*, 2025). *GREB1* expression is upregulated by progesterone and GREB protein modifies downstream regulation of genes by progesterone influencing endometrial decidualization and embryo implantation (Chadchan *et al.*, 2024). *GREB1* is also regulated by oestrogen in both mouse and human endometriotic cells, affecting oestrogen-mediated gene expression. Differentially spliced transcripts may play a role in regulating these feed-forward loops for progesterone and oestrogen receptor in normal endometrial physiology and endometrial pathology.

The lead SNP on chromosome 7 alters expression of *TRA2A* (Transformer 2 Alpha Homolog). *TRA2A* encodes a protein with RNA recognition and plays a role in RNA splicing (Rahmioglu *et al.*, 2023). On chromosome 8, lead SNPs associated with endometriosis regulate methylations signals and the risk allele decreases expression of *GDAP1* (Ganglioside Induced Differentiation Associated Protein 1) (Rahmioglu *et al.*, 2023). This gene is also associated with severity of dysmenorrhea and neuronal development (Barneo-Munoz *et al.*, 2015; Jones *et al.*, 2016).

At chromosome 9p21, the risk SNP identified in a Japanese population (Uno *et al.*, 2010) affects expression of *CDKN2B-AS1*, a long-noncoding RNA overlapping the Cyclin Dependent Kinase Inhibitor *CDKN2B* (Nakaoka *et al.*, 2016). The lead SNP in this Japanese study is population specific and lies in a cis-regulatory element that interacts with the promoter of *CDKN2A-AS1* or *ANRIL*, with allele specific expression of *ANRIL* in eutopic endometrial tissues and endometrial carcinoma cell lines (Nakaoka *et al.*, 2016). *ANRIL* regulates progression in the cell cycle including regulation of *CDKN2B* and modifies activity of multiple miRNAs involved in cellular processes including cell proliferation, invasion, and inflammation (Sanchez *et al.*, 2023). Dysregulation of *ANRIL* disrupts normal cell cycle control, leading to uncontrolled cell growth and *ANRIL* expression is upregulated in more than 20 types of cancer (Sanchez *et al.*, 2023). Subsequent GWAS studies in European populations identified an association between endometriosis and other variants at this region of chromosome 9p21 (Sapkota *et al.*, 2017; Rahmioglu *et al.*, 2023), but the functional role of these variants is yet to be determined.

On chromosome 12q22, the lead SNP lies in a bidirectional promoter with the risk allele increasing expression of both *FGD6* and *VEZT* in endometrium (Fig. 1) (Mortlock *et al.*, 2022). Both VEZT and FGD6 have roles in cell adhesion and cytoskeletal remodelling relevant to the development of endometriotic lesions (Guo *et al.*, 2011; Holdsworth-Carson *et al.*, 2016). *FGD6* is a member of a family of guanine nucleotide exchange factors regulating cellular development, plays a role in osteoclasts regulating the assembly of different actin-based protein networks, and activates another endometriosis candidate, *CDC42* (Steenblock *et al.*, 2014). *FGD6* is significantly overexpressed in gastric cancer samples, compared with adjacent normal tissues, and is associated with poor prognosis (Zeng *et al.*, 2021). *VEZT*, an adherens junction transmembrane protein, is a putative tumour suppressor in gastric cancer and can inhibit growth, invasion and tumorigenesis (Guo *et al.*, 2011; Miao *et al.*, 2013).

The lead SNP on chromosome 15 regulates methylation signals and the risk allele decreases expression of the Signal Recognition Particle 14 gene (*SRP14*) with additional evidence for chromatin interactions between regulatory regions for *SRP14* and the promoter of *BMF* (Bcl2 modifying factor) (Rahmioglu *et al.*, 2023). On chromosome 17, an associated risk SNP regulates expression of the *HOXB9* (Homeobox B9 HOX gene). *HOXB9* is a transcription factor influencing expression of genes related to organ development, tissue differentiation, and immune response (Hjazi *et al.*, 2024). The gene shows altered regulation in multiple malignancies, including increased expression in breast cancer associated with increased metastatic capacity and poor prognosis. Multi-omics analysis of the latest gene mapping results provides robust evidence for a role for *SEMA3F* in endometriosis pathogenesis and roles for *EEFSEC*, *UBE2D3*, *DTD1*, and *VEZT* in cellular morphogenesis associated with endometriosis (Koller *et al.*, 2026).

### Genetic regulation of epigenetic signals

Common genetic risk factors regulate epigenetic signals with roles in transcription and cellular programming. DNA methylation is the most widely studied epigenetic mark and can also be modified by exposure to environmental factors. Methylation signals in endometrial samples and their association with endometriosis were investigated in a large sample of 984 participants with genotype data (Mortlock *et al.*, 2023). Menstrual cycle phase was a major source of variation in methylation signals, suggesting cellular and hormonally driven changes across the cycle can regulate genes responsible for endometrial physiology and function. In this large study, no significant differences in the degree of methylation were detected for individual signals between all endometriosis cases and controls. Previously reported differences in methylation at candidate genes were not replicated, and this lack of replication may be due to use of eutopic versus ectopic tissue, small sample sizes used previously, and effects of variation across the menstrual cycle (Mortlock *et al.*, 2023). Four significant differences in DNA methylation signals were associated with severe (Stage III/IV) endometriosis, consistent with greater genetic effects on endometriosis risk in severe cases. Genes annotated to these differentially methylated sites include *EEF2* (eukaryotic translation elongation factor 2), which is downregulated in ectopic endometriosis lesions, and the nuclear transport receptor *TNPO2* (Transportin 2) highly expressed in the ovary, uterus, testis, and brain.

Analysis of genetic effects on methylation signals identified over 100 000 independent cis-mQTLs in endometrium, including 51 associated with risk of endometriosis (Mortlock *et al.*, 2023). Functional evidence for epigenetic targets contributing to endometriosis risk included methylation signals detected in predicted enhancers that interact with *GREB1* in the uterus and ovary. Two possible endometrial-specific mQTLs were also located in regulatory regions associated with the expression of endometriosis risk genes *LINC00339*, *CDC42*, and *WNT4* on chromosome 1p36.12. As noted above, endometriosis-associated mQTLs on chromosomes 8, 15, and 17 are strongly correlated with SNPs associated with endometriosis in each region that alter transcription of *GDAP1*, *SRP14*, and *HOXB9* (Mortlock *et al.*, 2023; Rahmioglu *et al.*, 2023).

Development of a methylation risk score aggregating effects across multiple DNA methylation sites and joint analyses with genetic risk factors (Thong *et al.*, 2025) demonstrated that the variance in endometriosis captured by both DNA methylation and common genetic variants combined (35%) was higher than estimates for either methylation (19.58%) and common genetic variants (28.83%) alone. The results suggest DNA methylation can influence endometriosis risk independent of genetic variation and further exploration in larger samples may shed light on how environmental exposures influence methylation and contribute to endometriosis risk (Thong *et al.*, 2025).

### Gene pathways

To date, there is functional support for only a limited set of genes regulated by genetic risk variants. In the absence of functional support, genes closest to the lead SNP can represent good proxies (Mostafavi *et al.*, 2023). Significant pathways from gene set enrichment analysis (Mootha *et al.*, 2003; Subramanian *et al.*, 2005) of genes implicated in endometriosis risk from the GWAS results and common somatic mutations include regulation of developmental processes involved in reproduction, reproductive system development, positive regulation of cell population proliferation, and epithelial cell differentiation (Table 2). Results for protein–protein interactions between endometriosis risk genes in these pathways show key genes in each pathway are also connected through reported protein–protein interactions (Fig. 2). The most recent GWAS results implicate pathways for cell differentiation, immune and hormonal regulation, tissue remodelling, and inflammation showing some overlap with previously reported pathways (Koller *et al.*, 2026). Many genes appear in multiple pathways, providing a mechanism where small genetic effects on regulation can be amplified by interactions within and between pathways. Thus, small genetic effects can have more profound effects on disease risk when several ‘hits’ are combined within common pathways.

**Figure 2.**
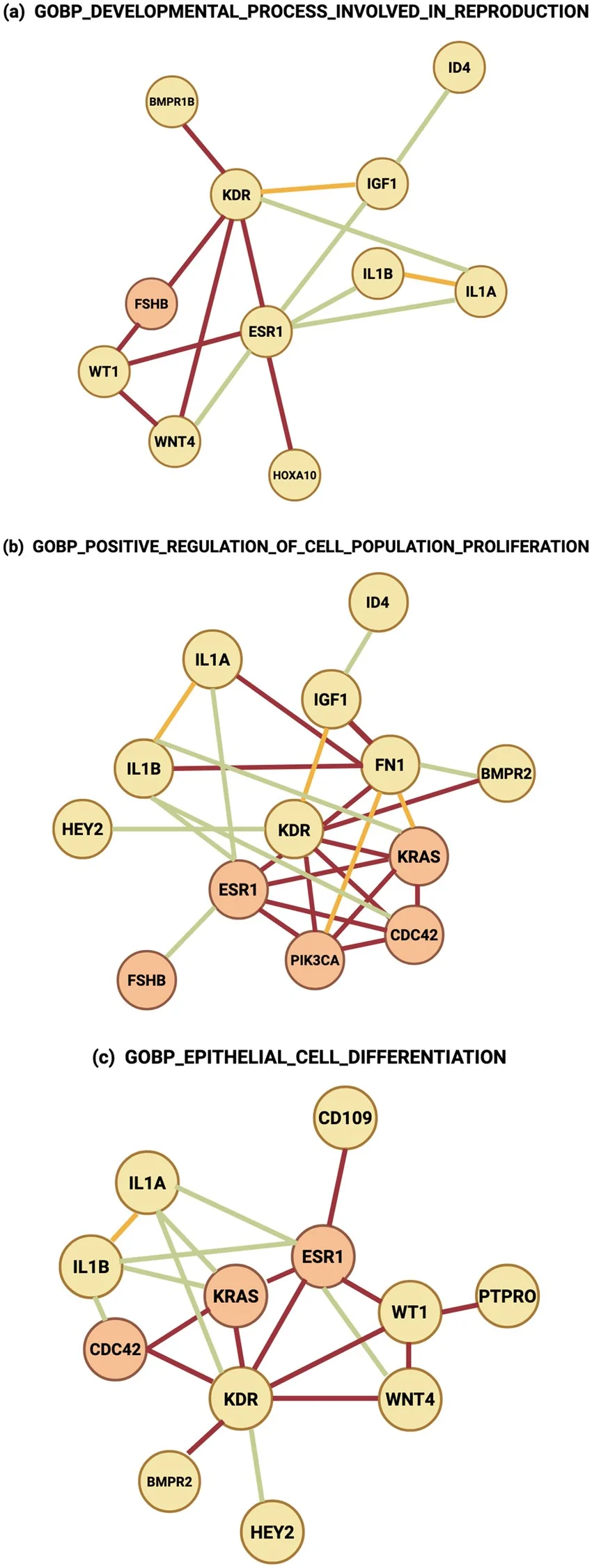
**Gene pathways implicated from genetic studies**. Interactions between genes for three of the top 10 pathways identified in gene set analysis showing protein–protein interactions evaluated in the STRING database (Szklarczyk *et al*., 2023) chosen from Table 2. The top 10 pathways included several related pathways and the pathways illustrated were chosen to show the top pathway GOBP_DEVELOPMENTAL_PROCESS _INVOLVED_IN_REPRODUCTION (Bonferroni adjusted *P*-value 2.65E-06); the most significant pathway for cell proliferation GOBP_POSITIVE_REGULATION_OF_CELL_POPULATION_ PROLIFERATION (adjusted *P*-value 4.06E-04); and GOBP_EPITHELIAL_CELL_DIFFERENTIATION (adjusted *P*-value 1.01E-03) given the importance of epitheal cells in endometriosis lesion development. Purple lines connecting genes denote experimentally determined interactions, orange lines denote interactions derived from curated databases, and green lines denote interactions based on prediction and co-expression. Genes symbols in pink circles denote a target gene with evidence that risk SNPs regulate gene expression (expression quantitave trait loci, eQTL) or genes with frequenct somatic mutations associated with endometriosis. Created in BioRender. Montgomery, G. (2026). https://BioRender.com/5wxetnd.

**Table 2.** The top ten pathways for Gene Ontology Biological Processes (GOBP) from Human Gene Set Enrichment Analysis following Bonferroni adjustment for multiple testing.

GeneSet	N^1^	n^2^	*P*-adj^3^	Genes
GOBP_DEVELOPMENTAL_PROCESS_INVOLVED_IN_REPRODUCTION	905	16	2.65E-06	WNT4, SLC19A2, IL1A, IL1B, BMPR2, KDR, ID4, ESR1, SYNE1, HOXA10, VPS13B, FSHB, WT1, IGF1, ACTL9, TEX11
GOBP_REPRODUCTIVE_SYSTEM_DEVELOPMENT	290	9	2.77E-04	WNT4, IL1A, KDR, ID4, ESR1, HOXA10, FSHB, WT1, TEX11
GOBP_POSITIVE_REGULATION_OF_CELL_POPULATION_PROLIFERATION	950	14	4.06E-04	CDC42, IL1A, IL1B, BMPR2, FN1, PIK3CA, KDR, ID4, HEY2, ESR1, FSHB, KRAS, HOXC10, IGF1
GOBP_DEVELOPMENT_OF_PRIMARY_SEXUAL_CHARACTERISTICS	219	8	4.86E-04	WNT4, IL1A, KDR, ESR1, HOXA10, FSHB, WT1, TEX11
GOBP_REGULATION_OF_CELL_POPULATION_PROLIFERATION	1702	18	5.55E-04	CDC42, NGF, IL1A, IL1B, BMPR2, FN1, PIK3CA, KDR, ID4, CD109, HEY2, ESR1, CDKN2A, FSHB, WT1, KRAS, HOXC10, IGF1
GOBP_EPITHELIAL_CELL_DIFFERENTIATION	712	12	1.01E-03	CDC42, WNT4, IL1A, IL1B, BMPR2, KDR, CD109, HEY2, ESR1, WT1, PTPRO, KRAS
GOBP_MALE_SEX_DIFFERENTIATION	161	7	1.06E-03	WNT4, IL1A, ESR1, HOXA10, FSHB, WT1, TEX11
GOBP_MULTICELLULAR_ORGANISM_REPRODUCTION	875	13	1.21E-03	WNT4, SLC19A2, IL1A, BMPR2, ESR1, SYNE1, HOXA10, VPS13B, FSHB, WT1, IGF1, ACTL9, TEX11
GOBP_POSITIVE_REGULATION_OF_CELLULAR_COMPONENT_ORGANIZATION	1041	14	1.25E-03	CDC42, WNT4, NGF, DNM3, IL1A, IL1B, BMPR2, FN1, PIK3CA, KDR, ESR1, IGF1, BMF, FRMD7
GOBP_REPRODUCTION	1418	16	1.50E-03	WNT4, SLC19A2, IL1A, IL1B, BMPR2, KDR, ID4, ESR1, SYNE1, HOXA10, VPS13B, FSHB, WT1, IGF1, ACTL9, TEX11

1 Total number of genes in the set.

2 Number of genes from the set also implicational in endometriosis development.

3 Adjusted *P*-value for the Gene Set analysis following Bonferroni correction. A target gene list including genes supported by clear functional evidence, genes nearest to the top risk variants from GWAS studies (Sapkota *et al.*, 2017; Rahmioglu *et al.*, 2023) and the three most common genes with reported somatic mutations was submitted to the GENE2FUNC module of the Functional Mapping and Annotation of Genome-Wide Association (FUMA) software to evaluate expression patterns and shared molecular functions between the genes (Watanabe *et al.*, 2017).

### Improving power for genotype–phenotype detection

Genetic studies on gene expression in endometrium have implicated some causal genes for endometriosis. However, the datasets available for endometriosis relevant tissues are based on small samples with limited statistical power for discovery (Burrows *et al.*, 2016; Fung *et al.*, 2018; Mortlock *et al.*, 2020). More generally, most GWAS ‘hits’ for complex diseases are not explained by known genetic effects on gene regulation (Umans *et al.*, 2021; Mostafavi *et al.*, 2023). Results from the GTEx Consortium suggest that only about 20% of GWAS hits colocalize with an eQTL in the GTEx dataset (GTEx Consortium, 2020).

This gap in our understanding of how disease risk factors modify gene regulation may be explained in part through genetic effects that alter RNA splicing, or context-specific effects acting only during development or in specific cell types. However, contributions from these effects so far are also modest (Mostafavi *et al.*, 2023; Yang *et al.*, 2025). Attempts to understand this knowledge gap show genetic risk factors for complex diseases are enriched in regulatory elements that are under strong negative selection and show complex regulation across different tissues and cell types (Mostafavi *et al.*, 2023). In contrast, most reported genetic effects on gene expression are variants with relatively large effects, often observed in multiple tissues and not subject to the same negative selection. These observations suggest current eQTL studies lack power to discover the gene regulatory mechanisms modified by disease causing variants which are more likely to be specific to individual tissues or cell types (Mostafavi *et al.*, 2023). More powerful multi-omics datasets, including genetic effects on gene expression (eQTL), splice variation (sQTL), methylation (mQTL), and protein expression (pQTL) at the individual cell level, will be necessary to speed up detection of disease risk genes and mechanisms.

Different methods are available to combine GWAS results with functional data to define risk genes. These different methods use distinct statistical frameworks and assumptions and results from the different methods are often inconsistent (Liu *et al.*, 2025). One approach to overcome these inconsistencies is to use a combination of the different methods to enable validation across different methods and window settings to improve accuracy and reproducibility (Liu *et al.*, 2025). Another limitation is that existing methods can integrate GWAS data with gene expression or epigenetic data but do not analyze multi-omics data simultaneously. Novel approaches to overcome this limitation include development of an integrated framework for joint analysis of GWAS and multi-omics QTL summary statistics (Wu *et al.*, 2023). Applying this framework to summary-level GWAS data for 50 complex traits showed that approximately 50% of GWAS signals are shared with at least one molecular phenotype. This framework is being extended to integrate GWAS and single-cell transcriptomic data to map trait-cell type associations (Song *et al.*, 2025). A recent study integrating large-scale endometriosis genetic risk data with single-cell transcriptomics, identified M2 macrophages as key drivers of disease susceptibility and implicated germline-regulated IL-1 signalling as a central, therapeutically actionable pathway underlying endometriosis pathogenesis and pain (Ochoa *et al.*, 2025). These findings motivate broader investigations that systematically extend GWAS and single-cell omic integration across cell types and disease stages to uncover additional causal cell types and pathways.

## Effect size is a poor predictor for the importance of target genes

The observed size of the genetic effects on disease risk are not good predictors of the effects of targeting genetically validated causal genes and pathways for disease prevention or treatment. Genetic support for a biological pathway provides important evidence for causality, and thus a potential for therapeutic intervention. Yet, this does not imply that the biological pathway can only be targeted through the specific genetic risk factors.

There are good examples where the effect size for a genetic risk factor is small, but a potent population-wide therapy has been developed. The risk variant at the dopamine D2 receptor gene (*DRD2*) locus with schizophrenia has a small genetic effect (risk ratio = 1.07). Despite this small genetic effect, dopamine D2 receptor antagonists can powerfully reduce schizophrenia symptoms revolutionizing care for individuals with schizophrenia, although incomplete efficacy and prominent side effects are acknowledged (Duncan and Deisseroth, 2024). Similarly, with hot flushes, the risk allele for neurokinin B receptor gene (*TACR3*) has a risk ratio of 1.12, but the highest dose of the neurokinin B receptor antagonist fezolinetant caused an 80.5% reduction in moderate/severe hot flushes after 4 weeks, and an 86.9% reduction in moderate/severe hot flushes after 12 weeks (Duncan and Deisseroth, 2024).

Genetic studies in endometriosis also demonstrate an important proof of concept of their value of defining critical pathways for future development of clinical treatments. Most drugs for primary medical management of endometriosis, including oral contraceptives, progestins and GnRH agonists target the reproductive endocrine system. Published GWAS studies (Rahmioglu *et al.*, 2023; Koller *et al.*, 2026) have identified multiple common genetic variants affecting endometriosis risk regulating genes within this system, *FSHB*, *ESR1*, *GREB1*, *BMPR2*, and a signal immediately downstream of *GNRH1* (Fig. 3). In addition, the risk SNP on chromosome 1 introduces an ESR1 (oestrogen receptor 1) binding site modifying hormonal responses in carriers of the risk allele (Zhang *et al.*, 2017). Together, these results provide strong genetic support for this pathway. Of course, development of drugs targeting the pathway was based on prior functional evidence and not genetic validation of these targets. However, this example of multiple genetic effects influencing the key pathway targeted by most medical treatments in endometriosis suggests much greater focus on functional follow-up of other genetic risk genes will help to define causes of endometriosis and identify novel drug targets.

**Figure 3.**
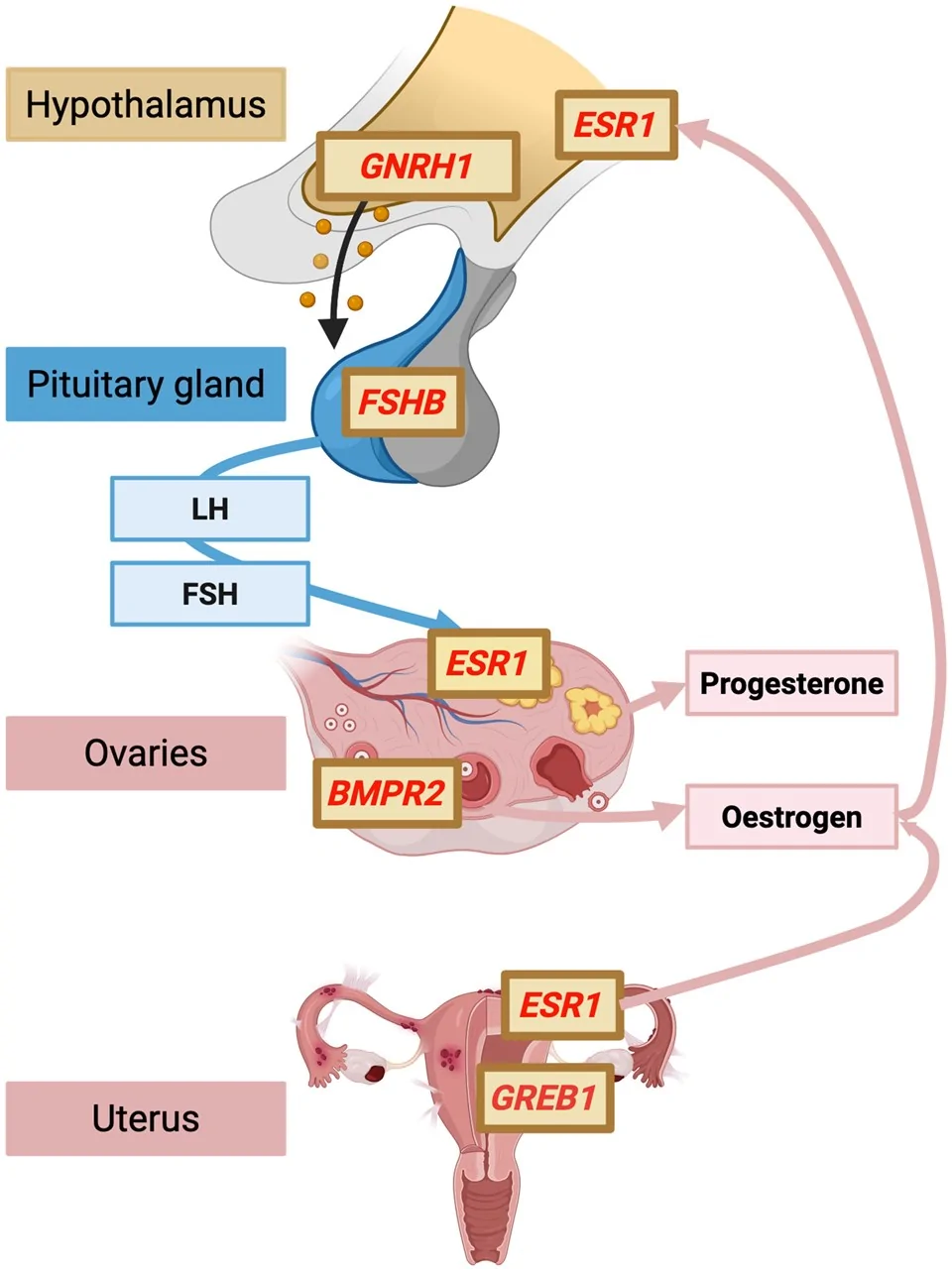
**Genes from the hypothalamic-pituitary-gonadal axis implicated from genome-wide association studies (GWAS)**. Genes from the hypothalamic/pituitary hormone signalling pathways regulating female reproduction implicated from GWAS for endometriosis risk are shown with red labels. The genes are all supported by the results of the recent International Endometriosis Genetics Consortium analysis (Rahmioglu *et al*., 2023). The figure was redrawn from a previously published figure that showed the hypothalamic-pituitary-ovarian axis with genes implicated in variation in twinning frequency and litter size (Montgomery, 2024). Created in BioRender. Montgomery, G. (2026). https://BioRender.com/1m3s7y9.

## The genetic architecture of endometriosis and comorbid conditions

Endometriosis is increasingly considered as a multi-system disorder with multiple comorbidities (Petraglia *et al.*, 2026). Discoveries from GWAS of many thousands of SNPs associated with multiple traits and diseases enable novel analytical approaches to address important questions about disease subtypes, molecular mechanisms, comorbidity, and causality (Visscher *et al.*, 2021). Genetic studies offer advantages for comorbidity studies because variation for individuals is defined at conception and the effects of disease-associated germline variants are less influenced by the course of a disease. Genetic risk for an individual and a given disease can be calculated from GWAS results and genetic overlap between traits is estimated by genetic correlation. Another design now widely used to understand how modifiable risk factors influence disease risk is Mendelian Randomization (MR) (Visscher *et al.*, 2021). In MR studies, genetic variants associated with a related disease or risk factor of interest (identified through GWAS) are analyzed to assess their effects on the disease outcome. Where there is genetic overlap, direction of causation can be assessed. MR also offers a cost-effective approach to test hypotheses before more formal and expensive clinical trials or when direct studies are not possible (Visscher *et al.*, 2021). Details of relevant methods and applications for genetic correlation and MR studies in endometriosis have been reviewed recently (McGrath *et al.*, 2023c), and these genetic approaches complement epidemiological studies, overcoming some issues of ascertainment bias.

### Genetic correlation between endometriosis and related traits

Individuals diagnosed with endometriosis are at increased risk of developing many comorbid conditions (Khan *et al.*, 2025). Leveraging large genetic datasets, the GRS for endometriosis is correlated with multiple conditions (e.g. excessive and frequent menstruation, uterine fibroids, urinary incontinence and asthma), reproductive characteristics (earlier age at menopause, shorter menstrual cycle length, earlier age at menarche, earlier age at first live birth), multiple pain traits, psychiatric conditions, cancer, urinary, bone, cardiovascular conditions, and blood/urine biomarkers (e.g. lower testosterone, higher triglycerides, higher calcium) in females in the UKB (McGrath *et al.*, 2023c). Some comorbidities can complicate diagnosis due to overlapping symptoms and can be affected by, or complicate, treatment plans (Rahmioglu *et al.*, 2023; Yang *et al.*, 2023; Pujol Gualdo *et al.*, 2025).

The genetic architecture of endometriosis and comorbidities can be leveraged to investigate whether a biological factor can explain each comorbid relationship. For traits genetically correlated with endometriosis, most correlations are moderate (McGrath *et al.*, 2023a,b; Rahmioglu *et al.*, 2023; Shigesi *et al.*, 2025). There are exceptions. Clear cell carcinoma, a type of epithelial ovarian cancer, has a genetic correlation of 0.71 (where a genetic correlation of 1 would indicate an identical genetic architecture) (Mortlock *et al.*, 2022). The genetic correlation with pain-related traits beyond the pelvic region provides evidence of more complex relationships between endometriosis and pain (Rahmioglu *et al.*, 2023). We expect the number of traits genetically correlated with endometriosis to increase as more well-powered GWAS data are generated and as phenotypes are better defined to reduce heterogeneity. Nevertheless, the range of correlated conditions across multiple biological systems implies that a diverse set of biological processes contribute to endometriosis risk.

The co-occurrence of endometriosis with other conditions could imply causal relationships, shared biological pathways, or spurious correlations owing to a confounding factor. For example, endometriosis is reported to be causal for uterine fibroids, benign growths in the myometrium of the uterus (Gallagher *et al.*, 2019). Although the disease-relevant tissue differs, hormone-signaling pathways are shared, which suggest the significant MR result is explained by shared driving mechanisms (Gallagher *et al.*, 2019).

### Shared genetic risk factors

Further characterization of the comorbid relationships includes fine mapping of the risk SNPs and annotating shared target genes. Co-localization analysis can determine the likelihood that a given risk locus contains a variant that is causal for both traits. This approach identified rs6025, a missense variant in the Factor V gene (*F5*), a variant identified as protective for endometriosis, diverticular disease, heavy menstrual bleeding and haemorrhoids (McGrath *et al.*, 2023a). The mutation reduces the deactivation of Factor V protein, a coagulation cascade protein. This variant may influence retrograde menstruation, given the association with heavy menstrual bleeding, since menstrual volume is likely correlated with the occurrence and volume of retrograde menstrual fluid. Shared genetic risk factors and pathways identified between endometriosis, osteoarthritis, and rheumatoid arthritis implicate immune signalling, extracellular matrix organization, and inflammatory processes (Shigesi *et al.*, 2025).

Malignant transformation of endometriosis to ovarian cancer was first suggested by Sampson (1924). MR approaches provide support for a causal effect of endometriosis on epithelial ovarian cancer, with strong histotype-specific effects evident from stronger causal effects for endometrioid and clear cell carcinoma histotypes (Yarmolinsky *et al.*, 2019; Mortlock *et al.*, 2022). For example, risk variants at the *SKAP1* locus were shared between endometriosis and most histotypes, whereas genomic regions near *KDR*, *GDAP1*, and *VEZT* were specific to clear cell ovarian cancer (Mortlock *et al.*, 2022). Discovery of endometriosis-subtype specific genetic variants may reveal further insights into the factors that promote and inhibit malignant transformation.

### Causal relationships between endometriosis and related traits

Recent MR studies identified an effect of lower testosterone levels on endometriosis risk (McGrath *et al.*, 2023b; Zhao *et al.*, 2023). Support for this influence of lower testosterone on endometriosis comes from evidence of association between endometriosis and shorter anogenital distances, a condition indicative of lower prenatal testosterone concentrations (Crespi and Dinsdale, 2024). Androgens are synthesized in the ovary and adrenal glands, control key functional processes in the endometrium including regulation of endometrial repair and proliferation when circulating oestrogens and progesterone are low (Simitsidellis *et al.*, 2018). Relative deficiency of androgens may affect endometriosis risk early in development and/or during reproductive life. Interestingly, low testosterone also appears to be causal for some histotypes of epithelial ovarian cancer, including clear-cell ovarian cancer (McGrath *et al.*, 2023b). This discovery opens a new research avenue to determine the mechanism(s) of low testosterone in promoting endometriosis and transformation to ovarian cancer.

There is evidence for causal effects of depression on endometriosis, but not the reverse relationship (Adewuyi *et al.*, 2021). Pathway-based functional enrichment analysis identified ‘abnormality gastric mucosa’ as one major overlapping pathway between the two conditions. Gastro-oesophageal reflux disease was subsequently identified as causal for, but not a consequence of, endometriosis, and to be bidirectionally causal with depression (Adewuyi *et al.*, 2021). This suggests endometriosis, depression, and gastric mucosa abnormalities are all causally related (Adewuyi *et al.*, 2021). A subsequent study demonstrated bidirectional causal relationships between irritable bowel syndrome and endometriosis, with overlapping genes including *MYSM1* (immune functions) and *FN1* (cell proliferation, motility, invasion, and migration) (Yang *et al.*, 2023). Recent sex-stratified GWAS have begun to shed light on the biological underpinnings of sex differences in complex disease (Sacks *et al.*, 2018). These findings are particularly relevant in the context of emerging evidence that sex-specific effects may influence conditions associated with endometriosis, including depression, potentially offering new insights into the interconnected pathophysiology of these conditions (Thomas *et al.*, 2025).

Genetic risk, quantified through GRS, has been shown to interact with comorbidities, amplifying endometriosis risk (McGrath *et al.*, 2025). For example, the presence of uterine fibroids increased endometriosis prevalence more markedly in women with a high GRS for endometriosis, suggesting that co-occurring conditions can modify the genetic predisposition to disease development. Complementary studies have used large-scale electronic health records and clustering techniques to identify distinct patient subgroups based on comorbidity profiles, ranging from anxiety and musculoskeletal disorders to allergies, anaemia, and migraines, highlighting the clinical heterogeneity of endometriosis (Sarria-Santamera *et al.*, 2023; Khan *et al.*, 2025). By integrating genetic data with clinical comorbidity profiles, we are beginning to unravel the biological mechanisms driving these interactions, knowledge that will inform more precise risk stratification and personalized care strategies tailored to individual disease trajectories.

Application of MR in complex disease genetics has increased dramatically in recent years. However, results should always be viewed with caution. Many submissions to journals are of dubious quality in part because of poor understanding of the methods among researchers and reviewers (Davey Smith *et al.*, 2026). Valid MR studies depend on explicit assumptions: genetic variants used must be associated with the exposure; there must be no common sources of confounding; and the variants must influence the outcome directly through the exposure and not via alternative pathways (McGrath *et al.*, 2023c; Davey Smith *et al.*, 2026). A common source of confounding is that genetic factors influence both an exposure and the outcome, and for many published studies, the notion that there are specific genetic influences on the exposures reported is simply implausible (Davey Smith *et al.*, 2026). Another important consideration is whether the eﬀect of the genetic variants on the disease of interest matches the eﬀect seen if the same exposure diﬀerence were produced through specific environmental changes. This is a fundamental principal of MR studies and known as gene-environment equivalence (Davey Smith *et al.*, 2026). Further research will be needed to determine the validity of each causal relationship and better understand the underlying mechanisms to provide the evidence base for better policy and improve clinical practice.

## Future directions

High priorities for research in endometriosis identified by patients and their families, clinicians, researchers, and in National Priority Statements (Horne *et al.*, 2017; Commonwealth of Australia, 2018; Giese *et al.*, 2023; Ellis and Wood, 2024) include understanding the causes of and risk factors for endometriosis and related issues of pain and infertility, improving diagnosis and treatments, and finding a ‘cure’. Results from genetic studies over the last 25 years address these priorities by discovering the genetic risk factors for endometriosis. Outcomes include mapping 80 genomic regions contributing to genetic risk, identifying candidate genes and pathways potentially involved in disease initiation and progression, elucidating the structure of genetic variation underlying the disease, discovering different genetic risk profiles for minimal/mild and severe disease, and demonstrating genetic overlap and potential causal relationships with comorbid conditions (Fig. 4).

**Figure 4.**
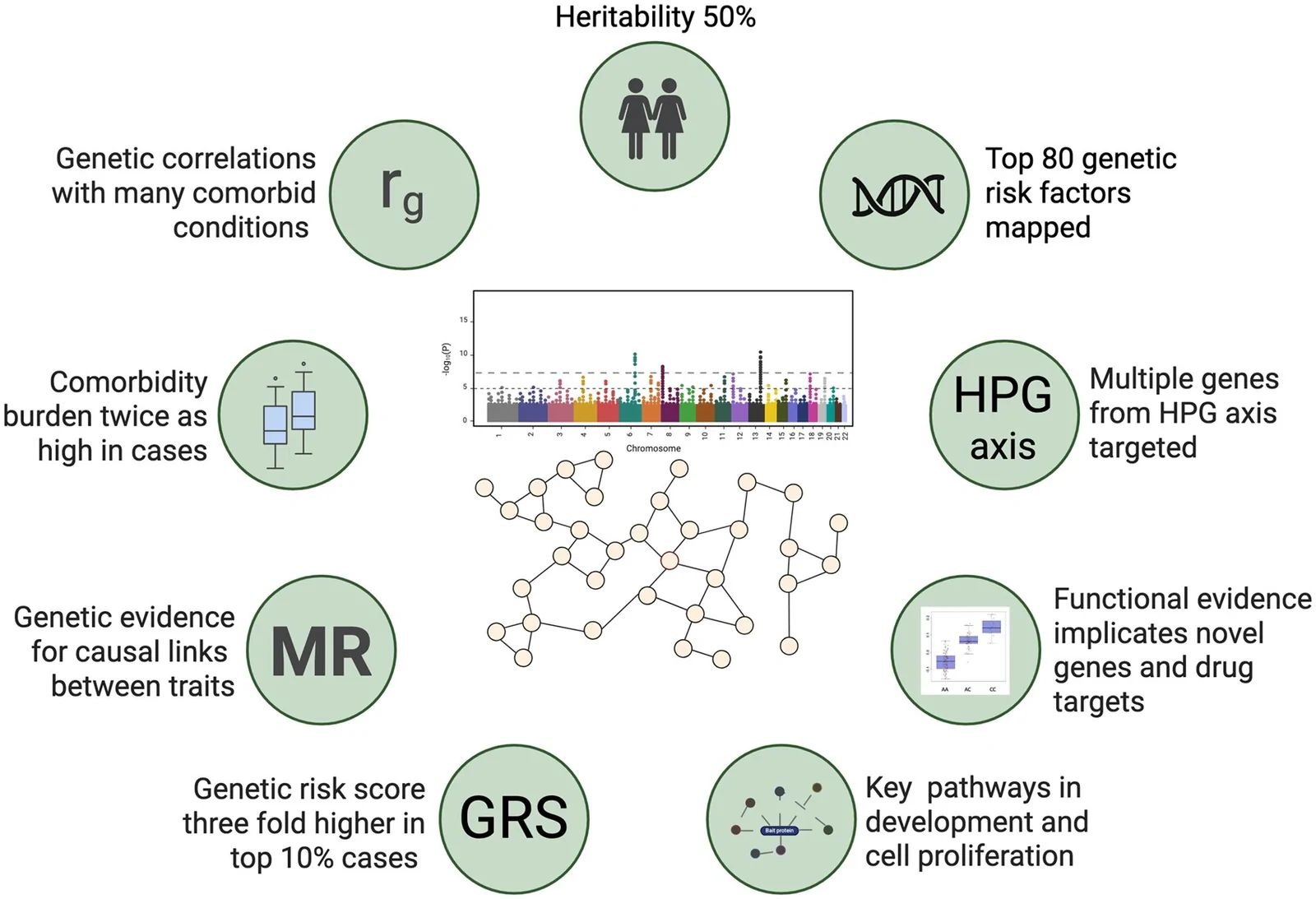
**Genetic contributions to understanding endometriosis risk**. Summary of the multiple outcomes from genetic studies of endometriosis risk. Abbreviations; genetic correlation (*r*_g_), Mendelian Randomisation (MR), genetic risk score (GRS). Created in BioRender. Montgomery, G. (2026). https://BioRender.com/bzqaf5p.

Future studies should address critical questions to enable more rapid translation and personalized treatments. One important question is the causes of disease heterogeneity and possible disease subtypes. The heterogeneity of endometriosis impacts diagnosis, clinical practice and the validity of much published research (Giudice *et al.*, 2023). Understanding whether disease subtypes exist is a complex question. Different types of lesions (e.g. superficial peritoneal, deep infiltrating endometriosis, or endometrioma) may represent subtypes, or there could be sub-types within lesion classes defined by specific molecular characteristics. Deep infiltrating endometriosis could result from progression of superficial peritoneal lesions or be a distinct entity. Endometriosis running in families (or associated with a high genetic burden) may be different from sporadic endometriosis (or endometriosis with low genetic burden). Multiple subtypes could coexist in one individual and could be dynamic, evolving from one to another as the disease progresses. Genetic differences associated with disease severity and lesion type support a case for distinct subtypes (Painter *et al.*, 2011; Sapkota *et al.*, 2015, 2017; Rahmioglu *et al.*, 2023). As noted earlier, future studies should include a greater proportion of participants with detailed clinical and phenotype data to better understand questions of heterogeneity as advocated for psychiatric disorders (Agrawal *et al.*, 2025). While the question of distinct subtypes is being settled, it is also critical that studies employ more robust designs that explicitly define their case samples or are large enough to adequately address the heterogeneity within patient groups and better account of the variation in this disease.

Progress towards understanding endometriosis pathobiology, as well as improving clinical management, would benefit from more effective integration of genetic evidence. The small fraction of variance in endometriosis risk explained by known genetic variants, together with the limited functional evidence linking these risk variants to disease mechanisms has led to questions about the utility of genetic research for this disease (Marquardt *et al.*, 2023; Endometriosis Initiative, 2024). However, genetic variation makes a substantial contribution to individual differences in endometriosis susceptibility. Mapped genetic risk factors point to multiple genes from the hypothalamic-pituitary-gonadal axis which remains the principal target for most drugs used in clinical treatment. Other risk regions likely implicate important pathways and the magnitude of genetic effect sizes for common diseases is not a reliable predictor of the therapeutic relevance of the pathways they implicate (Duncan and Deisseroth, 2024; Montgomery, 2024). Defining how genetic variants contribute to disease risk requires a multidisciplinary approach to prioritize the most promising candidates and employs a range of experimental approaches (Tapmeier *et al.*, 2021). Greater emphasis on genetically informed functional studies to identify causal genes, and molecular pathways will help elucidate disease mechanisms. Collecting genetic data together with clinical data and advanced imaging will also help understand the relationship between genetic risk, disease severity, and disease subtypes defined by different classification systems, and can exploit new opportunities from advanced imaging to track disease progression and evaluate genetic risk.

Functional genomic datasets are becoming available but remain incomplete, mainly derived from blood or post-mortem samples and often lack genetic data (Zeng and Visscher, 2025). Well powered studies with clinical data, genetic data, gene expression, epigenetics, single cell sequencing, spatial transcriptomics and proteomics across a range of cell types relevant to endometriosis and across diverse experimental and disease conditions will be required to define which risk variants have functional impact (Zeng and Visscher, 2025; Wray *et al.*, 2026). Spatial transcriptomics is beginning to show differences in function for the same cell-types in difference locations (Wray *et al.*, 2026) and integration of GWAS results with spatial transcriptomic data (gsMap) provides a large library of spatial transcriptomic images annotated with GWAS results for 110 complex traits (Song *et al.*, 2025; Wray *et al.*, 2026). International genetics studies have demonstrated the value of large collaborative efforts to combine data. Similar collaborative frameworks will benefit functional studies in endometriosis. Robust experimental designs that address specific research questions while enabling data integration across studies will be critical to establish datasets with sufficient statistical power to detect subtle regulatory effects underlying disease processes.

Environmental exposures also contribute substantially to the risk of endometriosis, so gene–environment interactions are probable, yet have not been investigated. Some genetic variants have detectable regulatory effects only under certain environmental exposures. This is observed for immune cells where variants controlling gene expression and methylation changes in response to glucocorticoid exposure are enriched for disease variants (Knauer-Arloth *et al.*, 2025) and 60% of colocalizations between genetic risk variants of immune-mediated disorders and CD4+ T cells were observed exclusively in their activated state (Soskic *et al.*, 2022). Despite endometriosis being considered an inflammatory condition, the role of the immune system in endometriosis development and progression is not completely clear, so this may be a relevant avenue to explore. Development of an integrated resource and cell atlas for genetic regulation of the epigenome, transcriptome, and proteome for relevant reproductive tissues and endometriotic lesions under various conditions would support functional studies and future translation.

Family history is included together with symptom data in some questionnaires designed to assess the likelihood of endometriosis and reduce time to diagnosis (Nnoaham *et al.*, 2012; Verket *et al.*, 2019; Chapron *et al.*, 2022; Mishra *et al.*, 2026). Genetic risk scores also capture genetic risk. These two estimates differ because the GRS is calculated from genetic markers while family history captures genetic risk plus effects of shared environment within the family. It will be important to determine which approach is best or whether both together can improve disease prediction. Studies on disease prediction should evaluate the use of symptom information and functional data in prediction models. Evidence on causal relationships between endometriosis and comorbid conditions should be expanded and evaluated for inclusion in clinical guidelines.

Genetic risk factors so far identified consist mostly of common variants with modest effect sizes that emphasize the complex nature of the disease. Understanding the role of somatic mutations and the search for rare coding variants are important avenues for future studies. A key focus of future studies in the Psychiatric Genetics Consortium (Agrawal *et al.*, 2025) is to examine the role of rare variants. Development of large data sets with whole-genome sequencing will provide greater power to detect the presence of rare variants contributing to endometriosis risk (Wainschtein *et al.*, 2026). These variants can directly identify the target genes and possible mechanisms of action that could speed up the process for the development of novel and more specific treatments for endometriosis. It is likely that common variants, rare variants, and somatic mutations combine to increase disease risk (Anglesio *et al.*, 2017; Montgomery *et al.*, 2020; Wainschtein *et al.*, 2026) and understanding all causes of variation will help understand disease biology and heterogeneity.

Genetic studies also help to establish the relationships between endometriosis and related conditions. As an example, leveraging the large genetic datasets available for psychiatric conditions has demonstrated widespread genetic overlap between related conditions challenging narrow definitions of specific disease diagnosis (Agrawal *et al.*, 2025). Results for endometriosis and other reproductive disorders are similar (McGrath *et al.*, 2023; Pujol Gualdo *et al.*, 2025) and clinical guidelines for endometriosis should consider the higher burden of comorbid conditions and their role in disease development and progression. The shared genetic risk factors for related conditions also provide insights that help explain effects on endometriosis risk and identify shared genetic risk factors.

Further, these shared genes and pathways may have pre-existing targeted treatments, which may prove useful in treating endometriosis. Repurposing previously approved drugs is an efficient strategy in drug development (Oskotsky *et al.*, 2024; Koller *et al.*, 2026) as the time and resources taken to demonstrate the drug is safe are significantly reduced, given that its safety profile has already been established in prior clinical trials. The comorbidities also provide preliminary evidence for subtypes of endometriosis. For example, uterine fibroids are more strongly associated with endometriosis on the ovary than endometriosis on the pelvic peritoneum, whereas infertility shows the opposite association (McGrath *et al.*, 2023c). As the availability of detailed clinical data grows, such endometriosis subtype-specific associations will become feasible to investigate (McGrath *et al.*, 2023a; Pujol Gualdo *et al.*, 2025).

## Summary and conclusions

The significant contribution of genetic risk factors to the development of endometriosis was first established 25 years ago. Despite a slow start in mapping specific risk factors, progress has accelerated dramatically with developments in technology, international collaborations to combine data and increase power, and access to other large genetic and genomics data resources. The top genetic risk factors have been mapped, and these implicate genes involved in development of the reproductive tract, the hypothalamic/pituitary/ovarian axis, regulation of cell proliferation, and regulation of epithelial cell differentiation. The studies also highlight important causal relationships between endometriosis and comorbid conditions with implications for clinical guidelines, a possible role of testosterone in disease development, and potential new drug targets demonstrating prospects for development of novel treatments.

In common with most other complex diseases, effects of individual common genetic risk factors are small. However, small effect sizes are not good predictors of the impact of drugs developed against genetically validated targets. Additive effects of small risk factors affect multiple genes in the same pathways and the fact that many potential risk genes act in multiple pathways may contribute to the effects of these genetic risk factors in disease initiation and progression. Genetic factors altering gene regulation may act in specific cell types and could have effects in different cells and tissues. Increasing access to cell-based models, single cell omics, spatial transcriptomics, and proteomics, together with genetic information and advanced statistical methods for multi-omics data integration will further accelerate progress.

Some aspects of progress from genetic studies are beginning to be included in clinical guidelines and future studies should address critical questions of heterogeneity and disease subtypes, expand the search for genetic risk factors to non-European populations, evaluate genetic prediction together with symptom data for early diagnosis and/or patient stratification, evaluate the role of rare and structural variants, and analyze large genetic datasets to better understand the causal relationships with comorbid conditions. Greater integration of insights from genetic advances into functional and clinical studies to understand endometriosis development and progression will help to accelerate progress towards earlier diagnosis and better treatments.

## Data Availability

No new data were generated for this review.
